# Formation of Nanopits in Si Capping Layers on SiGe Quantum Dots

**DOI:** 10.1007/s11671-010-9811-y

**Published:** 2010-10-02

**Authors:** Jian Cui, Jian Hui Lin, Yue Qin Wu, Yong Liang Fan, Zhenyang Zhong, Xin Ju Yang, Zui Min Jiang

**Affiliations:** 1 State Key Laboratory of Surface Physics, Fudan University, 200433, Shanghai, People's Republic of China

**Keywords:** Nanopit, Quantum dot, Capping layer, SiGe, Strain energy, Relaxation, Surface energy

## Abstract

In-situ annealing at a high temperature of 640°C was performed for a low temperature grown Si capping layer, which was grown at 300°C on SiGe self-assembled quantum dots with a thickness of 50 nm. Square nanopits, with a depth of about 8 nm and boundaries along 〈110〉, are formed in the Si capping layer after annealing. Cross-sectional transmission electron microscopy observation shows that each nanopit is located right over one dot with one to one correspondence. The detailed migration of Si atoms for the nanopit formation is revealed by in-situ annealing at a low temperature of 540°C. The final well-defined profiles of the nanopits indicate that both strain energy and surface energy play roles during the nanopit formation, and the nanopits are stable at 640°C. A subsequent growth of Ge on the nanopit-patterned surface results in the formation of SiGe quantum dot molecules around the nanopits.

## Introduction

Heteroepitaxy has been a powerful method to fabricate functional quantum structures, e. g. quantum wells [1], quantum dots (QDs) [2] and quantum rings (QRs) [3,4]. On the one hand, strain is the most important factor affecting the formation of nanostructures [1-3] and even their capping layers in heteroepitaxy [5]. The evolution of the strain can result in a variety of nanostructures, such as QRs [3]. On the other hand, strain induced by heteroepitaxy has been a very prominent improvement in technology to increase carrier mobility [6]. Recently, strained Si channel induced by SiGe QDs has been proposed to enhance hole mobility in field effect transistors [7]. Thus strain, together with its distribution and evolution is a key to understand the growth mechanism of the quantum structures and realize the desired structures.

Nanopits are interesting for their use as template to achieve positioning growth of QDs, [8-10] QRs [11] and QD molecules [12]. In 1998, Deng and Krishnamurthy [12] fabricated nanopits by depositing carbon impurity in Si matrix, in which SiGe QD molecules were grown around each nanopit. In recent years, nanopits fabricated by electron beam lithography, [8,13] holography lithography [9] and nanosphere lithography [10,11] have been used to fabricated ordered SiGe QDs or QRs. Recently, nanopits were also observed in III–V material system by self-assembling based on droplet epitaxy growth [14].

QD molecules are promising candidates as building blocks for the quantum computing [15-17]. It is highly desired to grow QD molecules on semiconductors for the possible applications in quantum computing. By self-assembly, in III–V system, GaAs [18], and InAs [19,20] QD pairs were grown by droplet epitaxy. In SiGe system, though the QD molecules has been observed by introducing carbon impurities [12], the growth of defect-free QD molecules needs to be further explored.

In this paper, a strain governed process, as well as related nanostructures in Si/Ge system are reported. Square nanopits, with a depth of about 8 nm and boundaries along 〈110〉, were formed in the low temperature grown Si capping layer by thermal annealing at 640°C. The formation mechanism is proposed based on strain energy relaxation and surface energy minimization. On the nanopit-patterned surface, by depositing Ge SiGe QD molecules were fabricated.

## Experimental procedure

The sample growth was carried out in a molecular beam epitaxy (Riber Eva-32) system with two electron beam evaporators of Ge and Si sources. P-type Si(001) substrates with a resistivity of 1–10 Ω cm were used to grow QDs. The substrates were chemically cleaned by Shiraki [21] method before put into load-lock chamber. After transferred into growth chamber, the substrates were heated to 980°C for 10 min to remove the protecting oxide, then clear 2 × 1 reconstruction pattern could be observed by reflection high energy electron diffraction. The chamber pressure was below 1 × 10^-9^ Torr. Then a 50 nm thick Si buffer layer was deposited at 650°C with a growth rate of 0.36 Å/s. At 640°C, by two-step growth method [22], two layers of Ge with a total thickness of 1.0 nm were deposited in sequence to form uniform QDs. The deposition rate was 0.11 Å/s. Then the sample was cooled down to 300°C to grow Si capping layers at a growth rate of 0.54 Å/s. The in-situ annealing process was performed at a high temperature of 640°C. After growth or annealing processes, the temperature was decreased to room temperature immediately. In order to unveil the detailed kinetics for the nanopit formation, in-situ annealing at a low temperature of 540°C was also carried out. Atomic force microscopy (AFM, Solver P47-SPM-MDT) was used to measure surface morphology. Cross-sectional transmission electron microscopy (TEM) was used to characterize microstructures.

## Results and discussion

**Figure 1:**
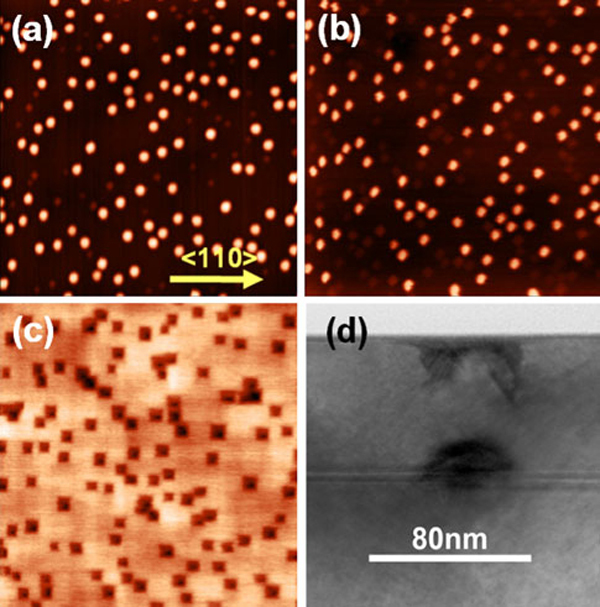
**AFM image (2 × 2 μm ^2^) of a the as-grown SiGe QDs, b the Si capping layer grown at 300°C with a thickness of 50 nm**. **c** the Si capping layer after in-situ annealing at 640°C for 10 min. **d** Cross-sectional TEM image of the sample shown in (**c**).

Figure 1a shows the surface morphology of the as-grown QDs. Two types of QDs, i. e. with shape of dome or hut, can be observed. The density of the dome-shaped QDs is 3 × 10^9^cm^-2^. The Si capping layer grown at a low temperature of 300°C on QDs shows similar surface morphologies [5] with a slight change in shape from dome to mound, as shown in Figure 1a and 1b. It is much different from the surface morphology of Si capping layer grown at a high temperature, i. e. the QRs with a thin Si capping layer and flat surface with a thick Si capping layer [3], since the surface migration of Si atoms and the interdiffusion between Si and Ge at this low temperature were inhibited greatly. After in-situ annealing at 640°C for 10 min, square nanopits were formed, as shown in Fig.1c. The surface morphology keeps unchanged with increasing annealing time. The nanopit density is almost the same as that of the as grown dome-shaped QDs, which indicate that the formation of the nanopits highly correlates to the QDs. Cross-sectional TEM image (Figure 1d) indeed shows that below each nanopit there exists one QD, confirming that the formation of the nanopits is associated with the buried SiGe QDs.

**Figure 2:**
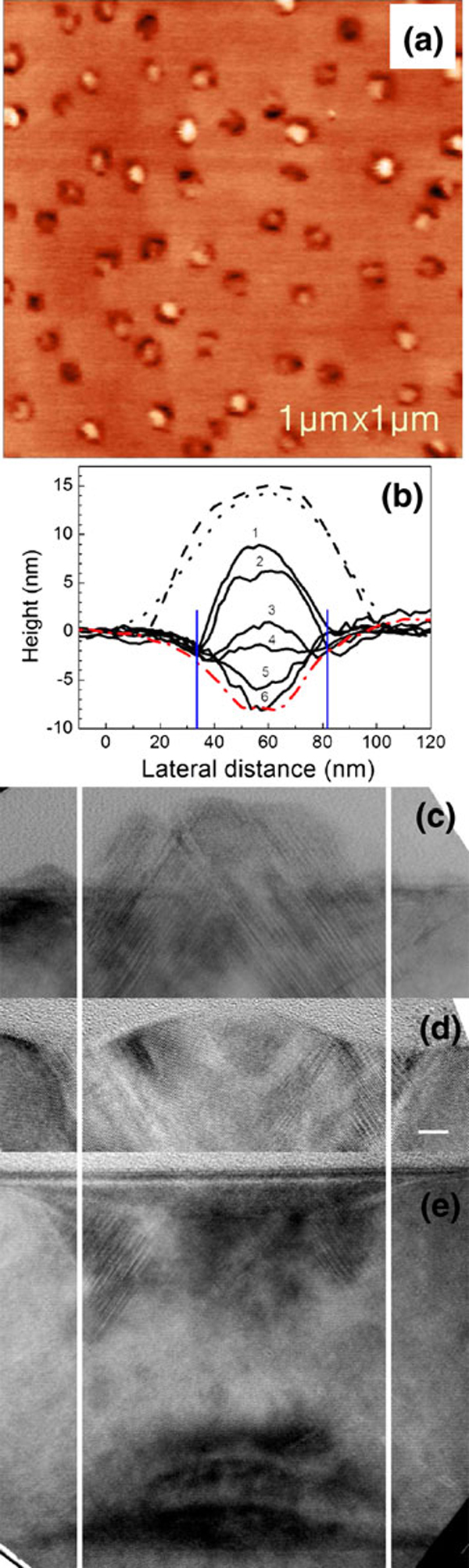
**a AFM image of the Si capping layer after in-situ annealing at 540°C for 7 min. b Cross-sectional profiles of an as grown QD (*dashed*), a Si mound before annealing (*dotted*), the transitional states from mounds to nanopits after annealing at 540°C for 7 min (*solid lines 1–6*), and a Si nanopit formed after annealing at 640°C for 10 min (*red dash dot*)**. Cross-sectional TEM images of **c** a Si mound before annealing, **d** a Si mound after annealing at 540°C for 7 min, and **e** a Si nanopit formed after annealing at 640°C for 10 min, the buried QD is also shown. The *scale bar* in (**d**) applies to (**c**) and (**e**). The *two blue lines* in (**b**) and *two white lines* in (**c**)–(**e**) indicate the deepest position of trenches.

In order to unveil the kinetic process of the nanopit formation, in-situ annealing was carried out at a low temperature of 540°C for a shorter period of 7 min (see Figure 2), which slows down all the atomic processes for the nanopit formation. Several typical profiles in transitional states from a mound to a nanopit are extracted from Figure 2a and plotted in Figure 2b. A large portion of the transitional states can be observed, which is due to the non-uniformity of the QDs, as well as the statistical fluctuation in the atomic processes. It is reasonable to take these profiles as the snaps at different evolutionary states, which are shown by lines 1–6 in Figure 2. From the evolutionary states, several features or conclusions can be drawn. (1) The Si atoms at the rims of the mounds together with the outer shell of the mounds may first migrate outwards, which is concluded by comparison of the profiles before annealing (dotted line in Figure 2b) and the ones in transitional states after annealing (solid lines 1–6 in Figure 2b). (2) After the initial migration, the migration of Si atoms at the rims tends to stop, and trenches around each mound are formed with a nearly constant depth of 2.5 nm. The deepest positions of the trenches are labeled by two vertical blue lines in Figure 2b and two white lines in Figure 2c–e. The distances between the lines are consistent with each other to be about 50 nm. (3) The final shapes of the nanopits keep nearly unchanged after further annealing.

Strain energy relaxation and surface energy minimization are considered as the two dominating factors for the nanopit formation. Firstly, atomic intermixing or interdiffusion between Si and Ge, and Ge surface segregation could be excluded during the process for the nanopit formation because the mounds consist purely of Si atoms and the capping layer is thick enough for ruling out Ge surface segregation (50 nm). The diffusion of Ge atoms in Si at the temperature lower than 650°C can be neglected. The whole process for the nanopit formation is only related to Si atomic migration.

**Figure 3:**
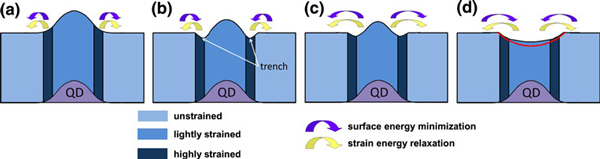
**Cross-sectional schematic diagrams of strain distribution in the Si capping layer and Si migration during annealing**. **a** At the beginning of annealing, both the strain energy relaxation and the surface energy minimization drive Si atoms at the rim migrate outwards. The Si atoms at the central part of the mound migrate outwards at a much slower rate. **b** When the surface at the rim is below the horizontal plane, the surface energy minimization drives Si atoms at the surrounding area to migrate inwards, the strain energy relaxation drives the Si atoms to migrate outwards. The trench is formed when the two opposite fluxes compensate each other. **c** After the trench is formed, the Si atoms at the central part still migrate outwards at a much slower rate driven by both strain energy relaxation and surface energy minimization. **d** When the surface area begins to increase, the surface energy minimization turns to induce Si to migrate inwards. When the inward Si flux induced by surface energy minimization compensates the outward Si flux induced by strain energy relaxation, the nanopit with well-defined shape is formed finally. The *red line* indicates the final profile of the nanopit. The *curly arrows* indicate the Si migration directions induced by strain energy relaxation or surface energy minimization.

Due to the existence of SiGe QD beneath the capping layer, the strain in the capping layer is tensile over the QD [23], compressive at the rim and null over the wetting layer. The strain energy gradient in the compressive region is much larger than that in tensile region [5], so the Si migration is faster at the rim than at the central part over the QDs. The migration processes for the Si atoms at the rim and at the central part may be distinguished as fast migration and slow migration, respectively. During annealing, the Si atoms at the rim migrate rapidly outwards onto the unstrained flat surface via the fast migration process, which results in the formation of the trench. At the same time, the Si atoms at the central part also migrate outwards via the slow migration process, which decreases the height of the mounds. Besides, the surface energy may play another important role due simply to the change in the surface area. At the beginning, the surface energy is also reduced due to the shrinkage of the surface area, which enhances the outward migration additionally, the scenario at this stage is depicted in Figure 3a. With the migration processing, when the surface at the rim is below the horizontal plane, the surface area and thus the surface energy turn to increase, inducing an inward flux, as shown in Figure 3b. As a result, the net outward migration of the Si atoms at the rim is reduced. When the inward flux induced by the surface energy minimization at the rim compensates the outward flux by the strain gradient, the trench is formed and in thermal equilibrium, as shown in Figure 3b.

**Figure 4:**
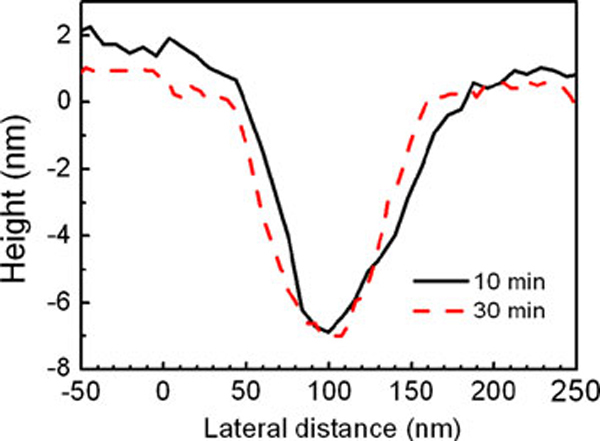
**Profiles of nanopits formed after annealing at 640°C for 10 and 30 min, respectively**.

After the trench is formed, the slow outward migration is still processing because the Si atoms at the central part of the mound is still strained. When Si atoms pass by on the surface of the trench, those atoms would experience a fast migration process due to the large strain energy gradient induced by adding Si atoms, and migrate quickly to the unstrained surface region. Thus the trenches maintain almost the same profiles during the slow migration process, as shown by lines 1–6 in Figure 2b, with all the lines merging to one at the trenches. Similarly, at the beginning, the surface energy minimization enhances the migration, as shown in Figure 3c. Likewise, with the migration processing, when the surface area begins to increase, the surface energy minimization turns to reduce the outward migration of the Si atoms, or induce an inward flux, as illustrated in Figure 3d. When the inward flux driven by the surface energy minimization compensates the outward flux by the strain energy gradient, the nanopits with well-defined shape are formed finally. If only the strain energy is considered, no inward flux exists, the nanopits will become deeper and deeper with annealing time. No significant difference can be observed between the profiles of the nanopits formed after annealing for 10 and 30 min at 640°C, as shown in Figure 4, further confirming that the formed nanopits are stable at the temperature of 640°C, and both strain energy and surface energy are responsible for the formation of the nanopits.

**Figure 5:**
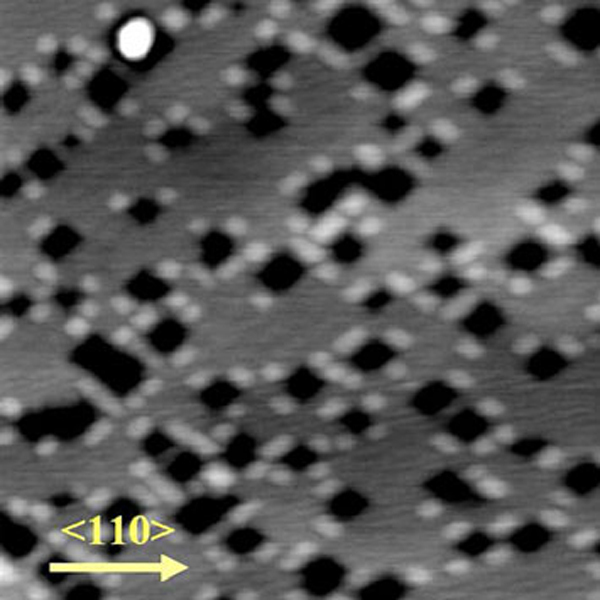
**AFM image (1 × 1 μm ^2^) of the QD molecules grown on the nanopit-patterned template**.

QD molecules are interesting for their applications in quantum computation. The fabrication of QD molecules is thus interesting for both fundamental research and practical applications. Here, we report a growth method of SiGe QD molecules on the nanopit-patterned surface formed by the process described above. Figure 5 shows the surface morphology of QD molecules by depositing 0.6 nm Ge on the nanopit-patterned surface at 650°C. The subsequently formed QDs are located at the sides around the nanopits. The boundaries are along 〈100〉 rather than 〈110〉 for the nanopits, which is induced by the preferential nucleation of Ge at the corners of the nanopit for smaller surface energy at the initial stage and subsequent strain energy relaxation during the islanding growth [12]. Compared with the work of Deng and Krishnamurthy [12], in which the nanopits are induced by carbon impurities, the remarkable advantage of the present work over theirs is that in the present growth process no other elements are involved, except Si and Ge. However, planar defects or stacking faults are formed in the capping layer in our case because the thickness of capping layer is larger than 20 nm, a critical thickness to generate stacking faults [5]. If the thickness of the capping layer is less than 20 nm, it is possible to grow defect-free QD molecules.

In the growth of ordered SiGe QDs, lithography technologies [8-10] are frequently used to fabricated nanopits on Si substrate. The grown QDs located in the nanopits, rather than around them, which is contrary to our results. The nucleation modes on the nanopit-patterned substrates have been observed and discussed based on the growth temperature [24]. They found that at a low growth temperature of 550°C, nucleation at the nanopits is in metastable phase. At high growth temperature of 750°C, QDs are stable at the terrace between nanopits. At a intermediate temperature of 650°C, random nucleation is observed. They attribute the variation of nucleation modes to the kinetic limitation at high growth temperature. In another theoretical paper [25], only random and ordered (in the nanopits) nucleation modes are predicted. In our experiments, the growth temperature is 650°C. Nearly all the QDs located around nanopits. However, by growing buffer layers with increasing growth temperature from 500°C to 640°C on nanopit-patterned substrates, QDs nucleate in the nanopits by growing at 640°C [11]. It can be deduced that different pre-deposited surface morphologies in the two cases result in different nucleation modes. It may be the key point that gradually increasing the growth temperature during the growth of Si buffer layers can effectively decreases the depth of the nanopits as well as the aspect ratio. Nevertheless, the morphological detail of nanopits plays important roles in determining the nucleation whether in or out of nanopits along with growth temperature.

## Conclusion

In summary, nanopits are obtained by in-situ thermal annealing of low temperature Si capping layer on QDs. The formation of the nanopits is discussed based on the strain energy relaxation and surface energy minimization. The strained Si mounds formed over the QDs become instable under thermal annealing, the Si atoms in the mounds migrate to the surrounding area and subsequently the nanopits are formed. The strain distribution in the Si capping layer defines the lateral size of the nanopits, which is close to the lateral size of QDs. QD molecules are grown by a subsequent deposition of Ge on the nanopit-patterned surface.
